# Supplementary material for: Bacterial lipids traverse the hydrophobic groove of TamB
Source: Biophys J. 2026 May 14;125(13):3379–89. doi: 10.1016/j.bpj.2026.05.019 (PMC13507460; doi:10.1016/j.bpj.2026.05.019)
Supplement: Supplementary file 2 [file mmc3.pdf]

Biophysical Journal
Article

# Bacterial lipids traverse the hydrophobic groove of TamB

Yiechang Lin[1,2,*] and Ben Corry[1]
[1]Research School of Biology, Australian National University, Canberra, ACT, Australia
[2]Present address: Center for Quantitative Biology, Peking University, Beijing, China

ABSTRACT The double-membrane envelope of Gram-negative bacteria protects them against environmental stress and antibiotics. Phospholipids are a core component of the bacterial outer membrane (OM). However, how phospholipids are transported to the OM from the inner membrane where they are synthesized is poorly understood. We show that the AsmA-like protein TamB transfers lipids through a large hydrophobic groove that directly bridges the inner and outer membranes. Lipid dissociation at the outer membrane is impeded when TamB is bound to its OM partner protein TamA, but lipids can spontaneously enter the groove in simulations where the C terminus of TamB is embedded in the OM. Other members of the AsmA-like family in *E. coli* can also accommodate lipids within their hydrophobic grooves, suggesting they may also function as lipid transporters. Our findings highlight the lipid transport ability of TamB and other AsmA-like proteins, suggesting their importance in maintaining bacterial OM integrity.

SIGNIFICANCE The outer membrane of Gram-negative bacteria is vital for survival, acting as a barrier against antibiotics and environmental threats. Yet part of this membrane is built from lipids made inside the cell, and how these lipids reach the outer membrane has been unclear. We show that the protein TamB facilitates lipid transport across the periplasm by forming a membrane-spanning tunnel. This mechanism resolves a major gap in our understanding of outer membrane biogenesis and suggests potential new strategies for disrupting bacterial defenses.

## INTRODUCTION

Gram-negative bacteria possess a double-membrane envelope that serves as a protective barrier against environmental stress and antibiotics.[1] This envelope comprises an inner membrane (IM) and outer membrane (OM) separated by an aqueous periplasmic compartment containing a peptidoglycan layer. The IM surrounds the cytoplasm and is composed of phospholipids, predominantly phosphatidylethanolamine, phosphatidylglycerol, and cardiolipin. The OM, which separates the periplasm from the external environment and plays critical roles in maintaining cell integrity, is highly asymmetric. Its outer leaflet is composed of lipopolysaccharides, which prevent the passage of hydrophobic molecules including antibiotics, while the inner leaflet is composed of phospholipids.

How intracellularly synthesized phospholipids are transported to the inner leaflet of the OM has eluded our understanding until recently, when members of the AsmA-like protein family were proposed as the conduits for lipid transfer from the IM to the OM.[2–5] In addition to AsmA-like proteins, other systems including the Mla pathway, Pqi system, and Let system have been implicated in phospholipid transport, with the Mla pathway proposed to mediate retrograde phospholipid transport, and the Pqi and Let systems suggested to form periplasm-spanning conduits for lipid transfer.[6–11] In *E. coli*, the AsmA-like family contains 6 members (TamB, YhdP, YdbH, YhjG, YicH, and AsmA), which are thought to be related to the eukaryotic repeating β-groove proteins that transfer lipids between organelles.[12] Deletion of the genes associated with the three largest members of this family (TamB, YhdP, and YdbH) generates a lethal phenotype, while deletion of *tamB* and *yhdP* together disrupts OM lipid homeostasis.[4,5] Furthermore, recent *in vivo* crosslinking assays and molecular dynamics simulations showed that phosphate-containing molecules directly interact with the hydrophobic groove of YhdP, and phospholipids can spontaneously enter the groove during simulations.[13] Results from these genetic, biochemical, and computational studies support the idea that TamB, YhdP,

*Submitted February 16, 2026, and accepted for publication May 12, 2026.*
*Correspondence: yiechang.lin@pku.edu.cn
Editor: James Gumbart.
https://doi.org/10.1016/j.bpj.2026.05.019
© 2026 The Author(s). Published by Elsevier Inc. on behalf of Biophysical Society.
This is an open access article under the CC BY license (http://creativecommons.org/licenses/by/4.0/).

and YdbH may act as redundant lipid transporters in *E. coli*, facilitating the spontaneous diffusion of phospholipids from the IM to OM.

The largest of these three proteins, TamB, complexes with the OM protein TamA to form the translocation and assembly module (TAM).[14] While the β barrel assembly machine (BAM) is responsible for catalyzing the assembly of most *E. coli* OM proteins, the structurally and evolutionarily related TAM has also been proposed to play a role in assembling several OM proteins.[14–17] Additionally, TAM was recently shown to directly catalyze the assembly of several OM proteins in a BAM-independent manner when reconstituted into liposomes.[18] These findings raise the question of whether TAM could play dual roles in transporting lipids and assembling OM proteins and what roles TamA and TamB each play individually during this process. Additionally, while previous studies imply a role for TAM in lipid homeostasis, it has been suggested that the phenotypes observed in these genetic studies could stem from secondary consequences of OM protein assembly,[17] and a direct role in lipid transport has yet to be conclusively shown.

Here, we use a combination of structural modeling and coarse-grained (CG) and all-atom molecular dynamics simulations to investigate the lipid transfer ability of TAM and other members of the AsmA-like family. Our data suggest IM lipids can spontaneously enter and traverse the large periplasmic spanning groove of TamB in a TAM model embedded in both the bacterial IM and OM. Lipid dissociation at the OM is impeded when TamA is in complex with TamB, but OM lipids are able to spontaneously enter the groove in simulations of TamB alone, implying a continuous pathway also exists for dissociation. Finally, we also demonstrate that all six members of the AsmA family in *E. coli* can accommodate lipids within their hydrophobic grooves. Our results support the idea that the TAM directly transports lipids from the IM to the OM and provide insights into the mechanisms through which this occurs.

## MATERIALS AND METHODS

### Coarse-grained molecular dynamics simulations

All CG simulations were prepared with the CHARMM-GUI Martini Bilayer Builder with the Martini 2.2 forcefield (unless otherwise noted) with elastic networks imposed (elnedyn22) and carried out using GROMACS 2023.[19]

CG simulations of full-length TAM, YdbH, and YhdP embedded in bacterial membranes were carried out to investigate their lipid transfer ability.

Simulations of the TAM complex were initiated from a structure produced by McDonnell et al. (2023) using a combination of AF2 predictions and membrane morphing simulations.[20] We removed residues 1–21 of TamA predicted to be a signal peptide by SignalP 6.0.[21] Two separate systems containing the TAM complex were generated and manually merged. The first system contained the N-terminal helix of TamB embedded in an 18 × 18 nm bilayer representing the bacterial IM with 75% POPE, 20% POPG, and 5% CDL2 in both leaflets. The total number of lipids in the IM is 1,040, 520 in each leaflet. The second system contained TamA embedded in an 18 × 18 nm bilayer (700 lipids total) containing 100% ReLPS in the outer leaflet and 90% POPE, 5% POPG, and 5% CDL in the inner leaflet to represent the OM. The total number of lipids in the OM is 700, with 200 ReLPS molecules (which have a larger area per lipid given the 4 tails in lipid A) and 500 lipids in the lower leaflet. The two systems were merged by aligning the protein complex and adding the IM lipids to the second system and removing the overlapping water molecules in the second system. A timestep of 15 fs was used in production simulations, and 5 replicates were carried out for 45 μs each.

To investigate whether lipid entry and traversal of the TamB groove can occur using an alternative structural model, we set up systems containing an unmorphed AF2 model of TamB embedded in a model IM as described above and simulated these systems in triplicate for 18 μs each.

In addition, to ensure that lipid entry and traversal were not an artifact of the double bilayer system and/or forcefield used, we carried out simulations with just TamB from the membrane morphed structure using the Martini 2.2 and Martini 3 forcefields. As Martini 3 CDL2 parameters have yet to be implemented in the CHARMM-GUI Bilayer Builder, we used an 18 × 18 nm membrane composed of 80% POPE and 20% POPG in these simulations, which were also carried out in triplicate for 18 μs each.

The predicted structure of *E. coli* YdbH (UNIPROT: P52645) was obtained from the AlphaFold Protein Structure Database.[22,23] The N-terminal helix was embedded into a symmetric bilayer mimicking the lipid composition of the bacterial IM as described above. A timestep of 20 fs was used in production simulations, and 5 replicates were carried out for 75 μs each.

The predicted structure of *E. coli* YhdP (UNIPROT: P46474) obtained from the AlphaFold Protein Structure Database contains an N-terminal loop that prevents unencumbered lipid access from the periplasmic leaflet of the IM. Prediction of the YhdP structure using AlphaFold2 (AF2) and AlphaFold3 (AF3) revealed that this loop is flexible (with multiple conformations present between predictions) and could be displaced from a blocking conformation by predicting YhdP together with palmitic acid molecules. For our simulations of YhdP, we thus used an AF3-predicted structure of YhdP in which the N-terminal loop was displaced using palmitic acid molecules. These molecules were removed prior to simulation and the N-terminal helix embedded into a bilayer mimicking the composition of a bacterial IM. A timestep of 20 fs was used in production simulations, and 5 replicates were carried out for 75 μs each.

Simulations of the C-terminal portion of TamB were carried out to investigate whether phospholipids spontaneously

enter from the OM. These simulations were initiated from a AF2 structural prediction of TamB (truncated, residues 900–1,259). A timestep of 20 fs was used in production simulations, and 3 replicates were carried out for 3 μs each.

Each system was solvated, neutralized, and ionized using 150 mM NaCl. Following the standard CHARMM-GUI protocol, the system was subjected to a short energy minimization, followed by a 5-step equilibration protocol spanning 4.75 ns in total in which restraints on the protein (1,000 kJ $mol^{-1}$ $nm^{-2}$, 500 kJ $mol^{-1}$ $nm^{-2}$, 250 kJ $mol^{-1}$ $nm^{-2}$, 100 kJ $mol^{-1}$ $nm^{-2}$, 50 kJ $mol^{-1}$ $nm^{-2}$) and lipid headgroups (200 kJ $mol^{-1}$ $nm^{-2}$, 100 kJ $mol^{-1}$ $nm^{-2}$, 50 kJ $mol^{-1}$ $nm^{-2}$, 20 kJ $mol^{-1}$ $nm^{-2}$, 10 kJ $mol^{-1}$ $nm^{-2}$) were gradually reduced. Production simulations were carried out in the NPT ensemble, with a pressure of 1 bar maintained using a Parinello-Rahman barostat with semi-isotropic conditions.[24] The temperature was maintained at 310 K using a v-rescale thermostat.[25] To prevent unrealistic protein deformation in the absence of an OM or OM anchoring region, we imposed weak backbone position restraints of 5 kJ $mol^{-1}$ $nm^{-2}$ in production simulations of all single bilayer systems.

## Structural predictions

To investigate the complexation interface between TamA and TamB and in search of a TamB structure that may plausibly span the periplasm alone, *E. coli* TamA, TamB, and TamA+TamB together (TAM) were predicted using Colabfold.[22,26] 100 structures (5 structures × 20 random seeds) were predicted in each case without relaxation or modification to the multiple sequence alignment, using 3 recycles.

To investigate whether the hydrophobic grooves of AsmA-like proteins are likely to accommodate lipids, the structures of *E. coli* TamB (UNIPROT: P39321), YhdP (UNIPROT: P46474), YdbH (UNIPROT: P52645), YhjG (UNIPROT: P37645), YicH (UNIPROT: P31433), and AsmA (UNIPROT: P28249) were each predicted with 50 molecules of palmitic acid using the AF3 online server.[27]

## All-atom simulations

All-atom molecular dynamics simulations were carried out using GROMACS 2023 with the CHARMM36 forcefield.[19,28] Simulation systems prepared using the CHARMM-GUI Bilayer Builder with three representative starting structures of TamB generated by AF2. These structures were truncated to include only the C-terminal portion (residues 900–1,259) and embedded in a membrane containing PVCL2, PMPE, PMPG, PVPE, and PVPG in a ratio of 2:8:1:8:2 in the lower leaflet and lipid A in the upper leaflet according to the protocol in Li et al. (2022).[29] The system was solvated, neutralized, and ionized with 150 mM NaCl. Following the standard CHARMM-GUI protocol, the system was subjected to a short energy minimization, followed by a 5-step equilibration protocol spanning 11.25 ns in total in which restraints on the protein backbone (2000 kJ $mol^{-1}$ $nm^{-2}$, 1000 kJ $mol^{-1}$ $nm^{-2}$, 500 kJ $mol^{-1}$ $nm^{-2}$, 500 kJ $mol^{-1}$ $nm^{-2}$, 10 kJ $mol^{-1}$ $nm^{-2}$), side chain (2000 kJ $mol^{-1}$ $nm^{-2}$, 1000 kJ $mol^{-1}$ $nm^{-2}$, 500 kJ $mol^{-1}$ $nm^{-2}$, 0 kJ $mol^{-1}$ $nm^{-2}$, 0 kJ $mol^{-1}$ $nm^{-2}$), and lipid headgroups (1,000 kJ $mol^{-1}$ $nm^{-2}$, 500 kJ $mol^{-1}$ $nm^{-2}$, 500 kJ $mol^{-1}$ $nm^{-2}$, 500 kJ $mol^{-1}$ $nm^{-2}$, 0 kJ $mol^{-1}$ $nm^{-2}$) were gradually reduced. Periodic boundary conditions were applied in all directions. The temperature was maintained at 310 K using the Nose-Hoover thermostat.[30] Production simulations used a timestep of 2 fs. Hydrogen bonds were constrained using the LINCS algorithm.[31] Electrostatic interactions were treated using the particle mesh Ewald algorithm with a cut off of 12 Å.[32] A van der Waals radius of 12 Å was used. Simulations were carried out in triplicate for 500 ns each.

## Analysis and visualization

Analysis scripts were written in python using the MDAnalysis, pandas, and scipy libraries.[33–35] All simulations were visualized and snapshots were prepared using Visual Molecular Dynamics (VMD).[36]

All analysis was performed on simulation trajectories post-processed to account for periodic boundary conditions, with the protein aligned to the first frame of the simulation. The trajectory was strided such that each frame represents 75 ns of simulation time for TamB simulations and 100 ns all other simulations. The z coordinates of lipid headgroups (PO4 beads) were tracked across the simulation time. The number of lipids present in the hydrophobic grooves of each protein was calculated as the number of headgroup (PO4, PO41) beads with z coordinates above the average z coordinate of the upper leaflet headgroups of the IM. Lipid headgroup and tail binding occupancies were calculated per residue by computing the proportion of simulation time for which any bead belonging to a phospholipid headgroup (PO4, GL0, GL1, GL2, NH3, PO41, GL11, GL21, PO42, GL12, GL22) or tail moiety (all other beads) could be found within 7 Å of the residue.

To calculate the cavity volume of YhdP, YdbH, and the morphed TamB model, we used the MOLEonline server to model the pathway through each protein, using "pore mode" with a probe radius of 45 and interior radius of 0.8 (parameters previously used to investigate YhdP).[13,37] We estimated the volume of each tunnel, which was represented by overlapping spheres, using a Monte Carlo integration method. Sphere centers and radii were extracted from the VMD script provided by the MOLEonline server. A bounding box encompassing all spheres was computed, and 10,000,000 random points were sampled within it. The tunnel volume was estimated as the bounding box volume multiplied by the fraction of points found inside at least one sphere. The length was calculated as the distance between the ends of the first and last sphere forming the tunnel. Pore radius profiles were generated by projection of the

sphere centers to the tunnel axis and plotting the corresponding radii as a function of position along the axis.

## RESULTS

### Inner membrane lipids spontaneously traverse the hydrophobic groove of TamB

To assess the ability of the TamA/TamB complex (hereafter referred to as TAM) to act as a conduit for lipid transfer from the *E. coli* IM to the OM, we carried out CG molecular dynamics simulations of TAM. Our starting TAM model was produced by McDonnell et al. using a combination of AF2Complex and membrane morphing simulations to generate a structure that can plausibly span the bacterial envelope.[20] In all replicate ($n = 5$) simulations of this model with the N-terminal helix of TamB embedded in the bacterial IM (modeled as 75% POPE, 20% POPG, 5% cardiolipin CDL2)[38–40] and TamA embedded in the OM (upper: 100% ReLPS, lower: 90% POPE, 5% POPG, 5% cardiolipin CDL2),[41] we observed lipids spontaneously entering the hydrophobic groove of TamB from the upper leaflet of the IM (Figures 1A and 1B, Video S1).

Over the course of each 45-μs simulation, lipids filled the wide, periplasm-spanning groove of TamB, traversing upward toward the OM. In several simulations (Figure 1B, replicates 1, 3, and 4; Video S1), lipids transiently dwelled approximately halfway up the groove before progressing further toward the OM, suggesting that this region may modestly slow lipid movement through the groove. By the end of the simulation, an average of 31.8 ± 1.5 lipids were present in the hydrophobic groove of TamB (Figure 1C). The relative proportions of lipid types within the groove closely matched the composition of the bacterial IM, with most of the lipids in the groove being POPE (Figure 1C).

While lipids moved freely from the IM into TamB, and lipids can pass each other within the groove, we did not observe any lipid dissociation events from the groove to the OM within the timescale of our simulations. Interestingly, lipids in TamB cease to move upward at around 10–20 Å from the OM (Figures 1B and 1D). This is due to the lack of clear hydrophobic path for the lipid as the hydrophobic groove of TamB ends (Figure 1D, inset; green helix, residues 1,137 to 1,150) before meeting the OM. An additional bent helix (Figure 1D, inset; purple helix, residues 1,160–1,185) occludes upward lipid movement in this replicate, presenting another obstacle to continuous lipid flow between the IM and OM via TamB.

As lipids moved through the hydrophobic groove (Figure 1E), their tail moieties interacted with other lipids in the groove as well as residues belonging to TamB for >95% of simulation time (Figure 1F, left). Protein and lipid interactions formed the major proportion of contacts for lipid tails (Figure 1F, right), with protein-tail interactions occurring on the interior of the TamB groove (Figure 1G). In contrast, the headgroups of these lipids predominantly interacted with surrounding water and the peripheral edges and loops of the protein (Figures 1F and 1G).

Spontaneous entry of inner membrane lipids into the hydrophobic groove of TamB was also observed in simulations of an unmorphed AF2 model of TamB embedded in a model IM single bilayer system (Figure S1A). This phenomenon was also observed for the morphed TamB model embedded in the IM in simulations using both the Martini 2.2 and Martini 3 forcefields (Figures S1B and S1C). This suggests that the entry of lipids into TamB is independent of the structural model, the use of a single or double bilayer system, and forcefield used in our simulations.

### AF2 predictions suggests alternative orientations of TamB in the OM

Given that free lipid dissociation at the bacterial OM does not occur in our TAM simulations using a structural model where TamA and TamB interact, we wondered if alternate configurations could allow free lipid exchange at the OM. One possibility is that the conformation of TamB within the TAM complex changes such that the hydrophobic groove moves closer to the lipid bilayer. To address this, we carried out AF2 structural predictions of TAM, TamA alone, and TamB alone, generating 100 structures of each using default parameters (without alteration of the multiple sequence alignment).

In all 100 predicted TAM structures, TamA and TamB interact at the beta barrel region, with additional interactions formed between the hydrophobic groove of TamB and the first polypeptide-transport-associated (POTRA) domain in TamA (Figure 2A). At the OM, beta sheet templating between residues 266–276 of TamA and the last 10 residues (residues 1,249–1,259) of TamB defines the interaction interface, consistent with a recently reported cryo-EM structure of the TamAB hybrid barrel.[42] The crystal structure[43] and AF2 predictions of TamA alone are similar (as evidenced by a template modelling score of 0.97), and an interaction between the beta strand formed by residues 266–276 and the final beta strand (residues 569–574) is seen in both structures, In contrast, predictions of the complex show a widening of the opening in the OM beta barrel due to interaction with TamB. All TAM structures adopted similar conformations, particularly in terms of the z-displacement of the hydrophobic groove of TamB relative to TamA (Figure 2B). As these structures were predicted without the context of the IM and OM, the resulting structures have lengths of around 100–150 Å, with some diversity sampled in the angle made between the IM helix (residues 1 to 28) and OM segments (residues 1,188 to 1,259) (Figure 2C). Notably, due to the lack of structural diversity in the produced TAM structures, none showed a hydrophobic groove

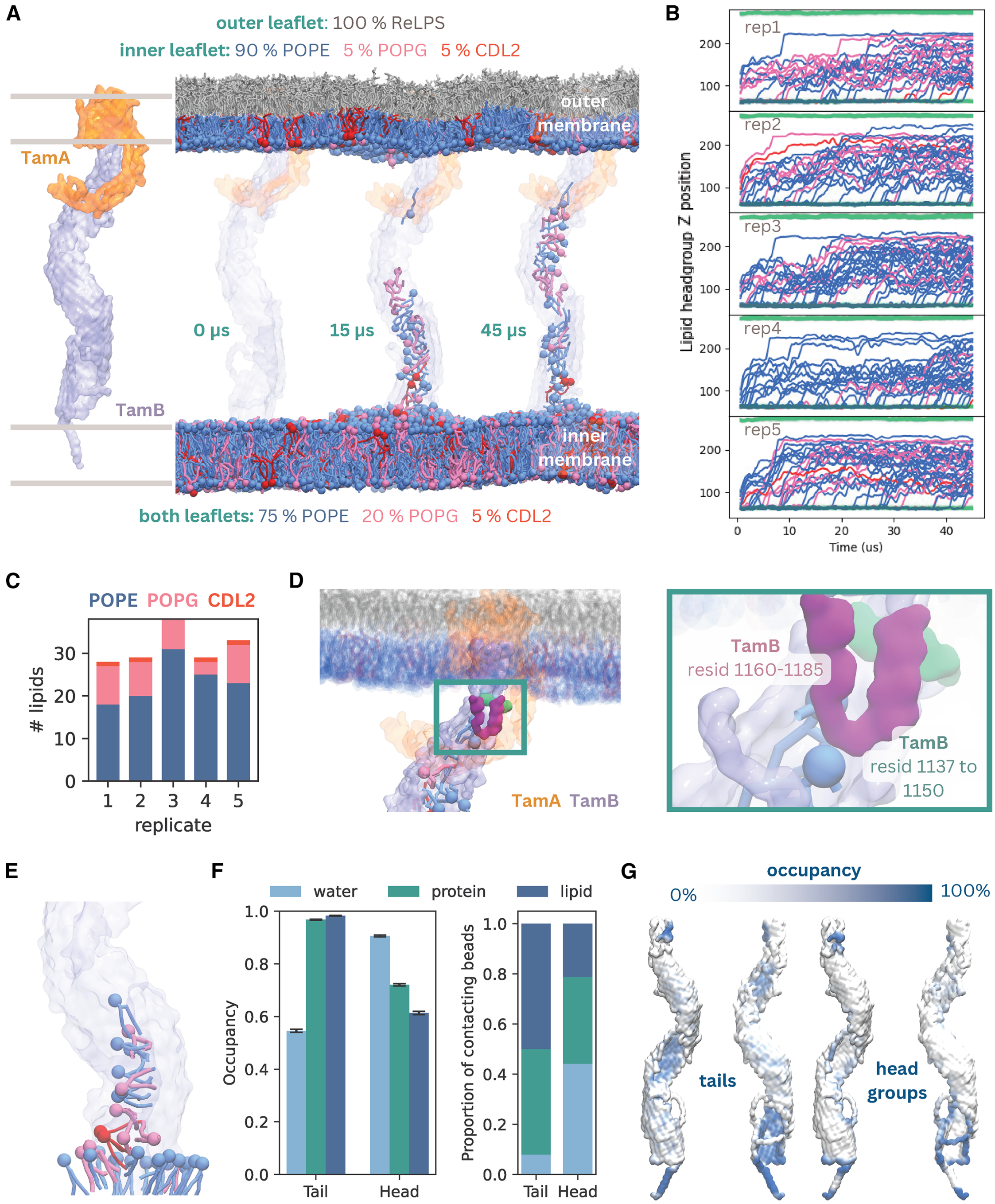


Figure 1. Inner membrane lipids spontaneously traverse the hydrophobic groove of TamB. (A) Structure of the TAM complex with TamA (orange) and TamB (purple) and their relative positions in the IM and OM (left).Representative snapshots of the TamA:TamB (TAM) complex embedded in the bacterial inner and outer membranes after 0, 15, and 45 μs of coarse-grained molecular dynamics simulations showing lipid movement from the IM into the hydrophobic groove of TamB. Lipids are colored by type (ReLPS, gray; POPE, blue; POPG, pink; CDL2, red). (B) Lipid headgroup z positions across simulation time, with each panel representing one of five replicates. Each line indicates the z position of one lipid headgroup, colored by the lipid type (POPE, blue; POPG, pink; CDL2, red). The green lines indicate the headgroup positions of lipids in the inner and outer membranes. (C) The number of different lipids present in the hydrophobic groove of TamB in each of five replicates after 45 μs of simulation, colored by the lipid type (POPE, blue; POPG, pink; CDL2, red ). (D) Representative snapshot after 45 μs of simulation, showing a POPG lipid blocked from moving further upward by a TamB helix (residues 1,160–1,185, purple), near the interface of TamA (majority of protein embedded in the OM, visible domains indicated in transparent orange) with TamB. A short helix (residues 1,137–1,150, green) in TamB marks the top of the hydrophobic column, which ends prior to meeting the OM. (E) Simulation snapshot showing lipid orientation as they enter the hydrophobic groove from the IM. (F) Contact occupancies (left) and proportion of contacting beads (right) for lipids in the groove (separated by tail and headgroup) with water, protein, and other lipids (error bars represent SEM, $n$ = 5). (G) TamB surface colored by occupancy of lipid headgroups (left) and tails (right). Regions of higher occupancy are indicated by darker coloring.

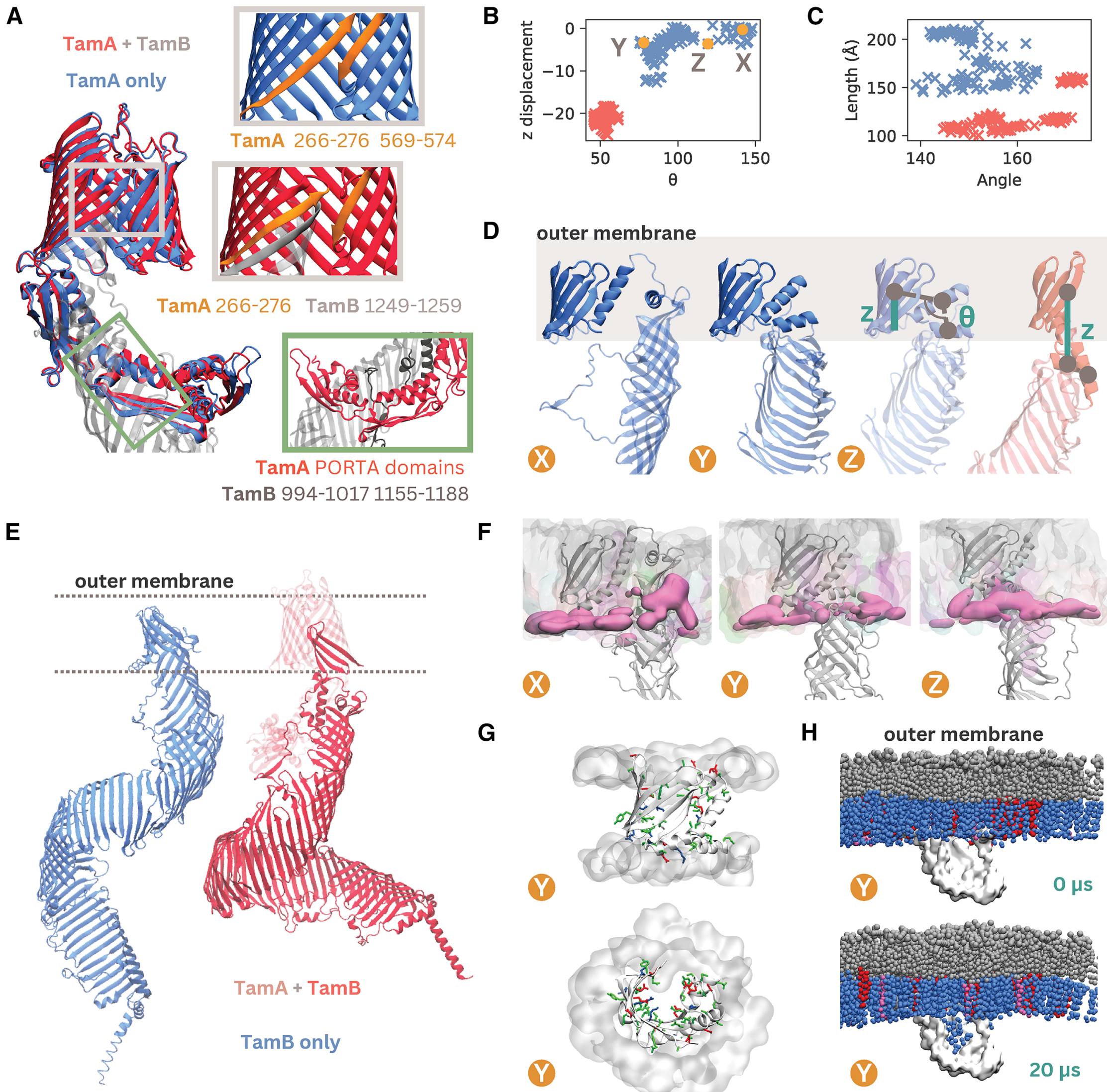


Figure 2. AF2 predictions suggest alternative orientations of TamB in the OM. (A) Overlay of the top-ranked AF2 structures of TamA, predicted alone (blue) and together with TamB (red). Inset: the beta strand interface of TamA predicted alone (top), showing an interaction between two beta strands (orange, residues 272–276 and 569–574) and TamA predicted together with TamB (middle), showing an interaction between a beta strand in TamA (orange, residues 266–276) and in TamB (gray, residues 1,249–1,259). Additional interactions are formed between the POTRA domains of TamA and residues 994–1,017 and 1155–1,188 of TamB (bottom). (B) The angle made by the beta strand region (residues 1,188 to 1,259), OM helix (residues 1,151 to 1,187), and helix at the top of the hydrophobic groove (residues 1,140 to 1,150) plotted against the z distance between the center of masses of the beta strand region and the upper region of the hydrophobic groove. Each datapoint represents one of 100 TamB structures predicted alone (blue) or together with TamA (red). (C) The angle made between the IM helix (residues 1 to 28) and the OM beta strand region (residues 1,188 to 1,259) plotted against the length of TamB in each predicted structure as measured by the distance between the center of masses of the IM helix and the OM beta strand region. Each datapoint represents one of 100 TamBs predicted alone (blue) or together with TamA (red). (D) Structural comparison of three representative structures of the OM portion of TamB predicted alone (blue) and with the OM portion of the top ranked TamA (red) with the metrics plotted in (C) indicated on the figure. The structures are labeled X, Y, and Z to correspond with the positions in (B) and reflect the structures presented in (F), (G), and (H). (E) The top-ranked AlphaFold2 structures of TamB, predicted alone (blue) and together with TamA (red, TamA shown in transparent coloring). (F) Representative snapshots from all-atom simulations of the three TamB structures embedded in the bacterial OM after 500 ns of simulation. The protein is shown using cartoon representation, with the lipid membrane depicted in a transparent surface. The locations of the phosphate atoms of lipids belonging to the lower leaflet of the OM are highlighted using a pink surface. (G) Snapshot of the TamB beta sheet region embedded in the OM during all-atom simulations viewed from the plane of the membrane (top) and from the extracellular space (bottom). Polar side chains are shown (red, acidic; blue, basic; and green, polar), and the location of the lipid headgroups near the protein is indicated by the gray surface. (H) Representative snapshots of TamB embedded in the bacterial OM after 0 and 20 μs of coarse-grained molecular dynamics simulations showing lipid movement from the OM into the top of the TamB hydrophobic groove.

that could be plausibly embedded in the OM to provide a continuous pathway for lipid dissociation.

Interestingly, structures of TamB differed considerably when it was predicted alone compared with predictions in complex with TamA (Figures 2B–2E). The longest predicted structures of TamB alone are ∼215 Å in length, making them likely to be capable of spanning the periplasm independently (Figure 2C). The absence of TamA in the prediction also leads to a significant change in the relative orientations of the OM beta strand region (residues 1,188 to 1,259) and OM helix (residues 1,151 to 1,187) as well as the hydrophobic groove (Figures 2B and 2D). This change in orientation (which would be sterically occluded in the presence of TamA) significantly alters which regions of TamB that would be embedded in the OM (Figure 2D), with the structures predicted in the absence of TamA placing the hydrophobic groove closer to the OM.

Given the differences between structural predictions of TamB in the presence and absence of TamA, we wondered whether the altered orientation of the OM portion of TamB when predicted alone may represent a physiologically relevant conformation through which phospholipids can freely move between the protein and the OM. To this end, we first assessed whether the alternate conformations of TamB could stably embed in the OM, especially as there are several polar and charged residues in portions of the protein predicted to be embedded in the membrane. To do this we selected three distinct representative structures (Figures 2C and 2D) and used all-atom simulations to assess the feasibility of this region to remain in the OM. After 500 ns, we observed some distortion in the lower leaflet of the OM in simulations using the top-ranked (by AF2, based on average pLDDT value) structure of TamB (X) due to the high placement of the hydrophobic groove, which has a polar outer surface (Figure 2F). However, this membrane distortion was not present in simulations using structures (Y) or (Z) where the groove was predicted to be slightly lower relative to the OM, and the models showed moderate root-mean-square deviation fluctuations while remaining stably embedded in the membrane (Figure S2). In these stable conformations, the C-terminal beta sheet residues 1,188 to 1,259 of TamB span the membrane, placing acidic residues at each end of the barrel among the lipid headgroups in each leaflet. Polar and charged residues in the middle of the sheets point inward in a partial barrel interacting with each other or water molecules, leaving a more hydrophobic surface to associate with the lipids (Figure 2G).

Subsequent CG simulations of structure (Y) showed that phospholipids belonging to the lower leaflet of the OM can spontaneously enter the top of the TamB hydrophobic groove. While this is opposite to the proposed physiological transport direction, it highlights the lack of a physical barrier for lipid dissociation in this region. This is not the case in simulations of TAM (as discussed above) nor in simulations of TamB predicted together with TamA, with TamA removed prior to simulation (Figure 2G). This suggests that lipid flow is not occluded by TamA directly but that when bound, it alters the conformation of TamB with respect to the OM.

Taken together, our data suggest that significantly different conformations of the TAM complex from those predicted here would be required to allow free lipid transfer to the OM in the TAM complex. In addition, our data suggest that TamB could span the periplasm without the aid of TamA and may possibly be oriented in the membrane in a manner that allows it to independently facilitate lipid flow, if correctly trafficked to and embedded in the OM.

## AsmA-like proteins may share the ability to conduct lipids

To assess how lipid transfer may differ between TamB and other members of the AsmA family in *E. coli*, we carried out 75-μs CG MD simulations of AF2-predicted structures of YhdP and YdbH, the two other members of the family suggested to transport lipids. For YhdP, the predicted structure on the AF2 database contained a flexible loop near the IM that completely prevented lipid entry in our simulations, so we carried out additional simulations using an AF3 model of the protein predicted with palmitic acid in the hydrophobic groove that caused displacement of the loop. In these simulations, we observe lipids spontaneously entering the hydrophobic groove of both YhdP and YdbH from the periplasmic leaflet of the IM (Figures 3A and 3B).

The number of lipids present in the hydrophobic groove differed between TamB, YdbH, and YhdP (Figure 3C), which may be due in part to the difference in cavity volume and length between these proteins (Figure S3A). As the protein with the widest hydrophobic groove and largest cavity volume (Figure S3), TamB was able to accommodate more than double the number of lipids when compared with YdbH and YhdP. During the 75-μs-long simulations, lipids fully traversed the groove of YdbH and reached the top of the protein. In contrast, owing to the narrow constriction in the YhdP groove (Figure S3B), lipids were prevented from fully occupying the length of YhdP. We note that the shorter length of YdbH, which does not span the periplasm alone, and the presence of the constriction in YhdP (Figure S3B), partially accounts for the reduced number of lipids within the groove when compared with TamB.

The proportion of each lipid type found within the grooves of TamB, YdbH, and YhdP mostly matched the composition of the IM (Figure 3D), although a few cardiolipin molecules were found in the grooves of TamB and YdbH, and none entered YhdP in any replicate.

Finally, given that AF3 was able to correctly place palmitic acid within the hydrophobic groove of YhdP, we carried out predictions for all 6 AsmA proteins with 50 palmitic acid molecules and showed that these are placed within the

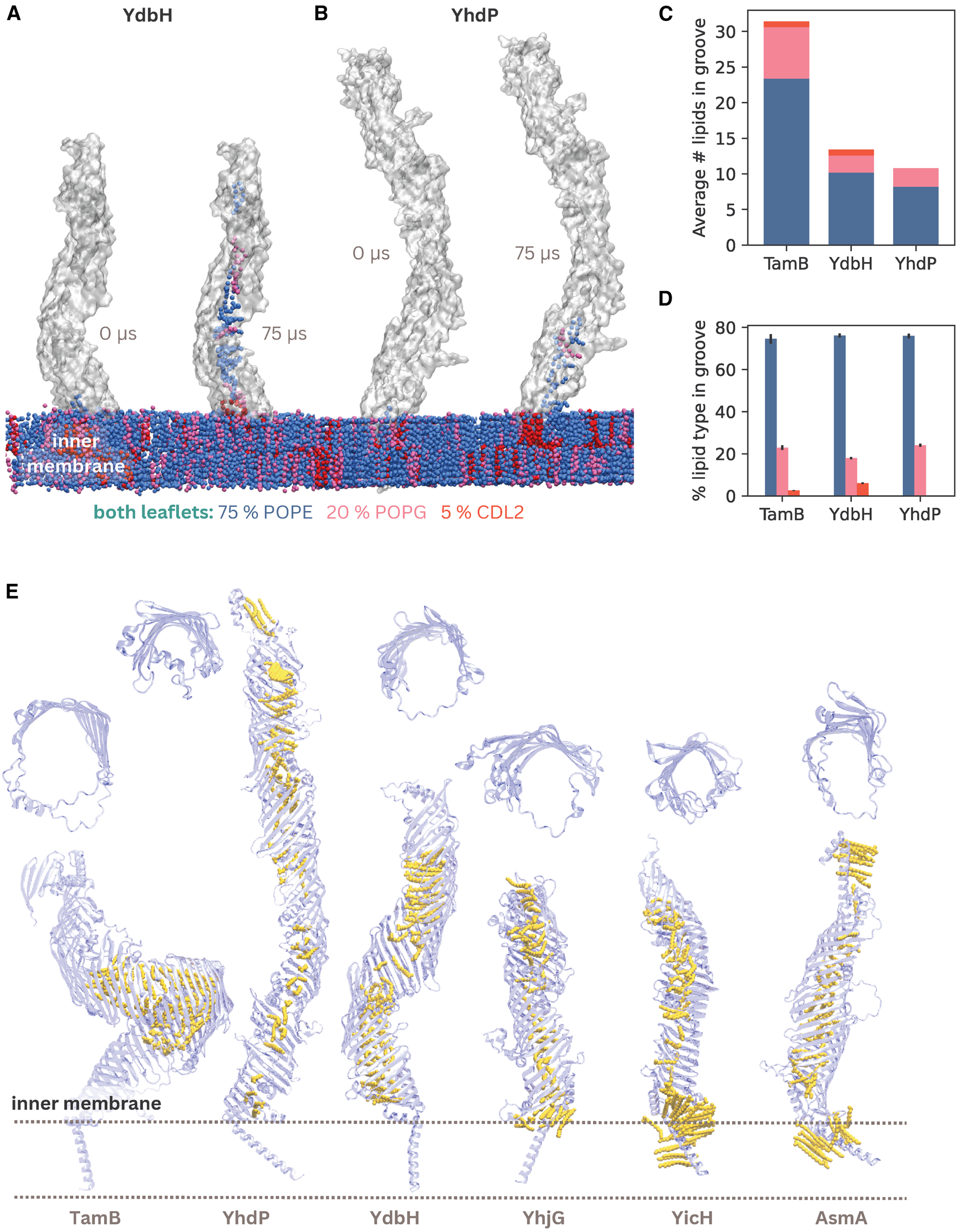


Figure 3. MD simulations and AF3 predictions of AsmA proteins suggest they share the ability to conduct lipids. (A) Representative snapshots of YdbH embedded in the bacterial inner membrane after 0 and 75 μs of coarse-grained molecular dynamics simulations showing lipid movement from the IM into the hydrophobic groove. (POPE, blue; POPG, pink; CDL2, red). (B) Representative snapshots of YhdP embedded in the bacterial inner membranes after 0 and 75 μs of coarse-grained molecular dynamics simulations showing lipid movement from the IM into the hydrophobic groove. (C) The average number of different lipids present in the hydrophobic groove of TamB, YdbH, and YhdP after 75 μs of simulation, colored by the lipid type (POPE, blue; POPG, pink; CDL2, red). (D) The percent lipid composition (error bars represent SEM, $n = 5$ for TamB, $n = 3$ for YdbH and Yhdp) within the hydrophobic groove of TamB, YdbH, and YhdP in the final frame of simulation, colored by the lipid type (POPE, blue; POPG, pink; CDL2, red ). (E) Snapshots of TamB, YhdP, YdbH, YicH, YhjG, and AsmA AF3 predictions made in the presence of 50 palmitic acid molecules (yellow). Cut-through images of each protein are shown above the full structure to indicate the width of the hydrophobic groove.

grooves of each protein (Figure 3E). Taken together, these data suggest that the hydrophobic groove of all 6 proteins can accommodate lipids.

## DISCUSSION

The AsmA-like family of proteins were recently proposed to act as conduits for lipid transfer between the bacterial IM and OM. One member of this family, TamB, interacts with an OM protein TamA to form the TAM. TAM is known to aid in the assembly of several OM proteins,[14–18] in addition to being implicated in lipid transport.[4,5] We sought to directly investigate whether TAM transports lipids and to elucidate the individual roles of TamA and TamB in this process. In simulations, phospholipids spontaneously enter and traverse the large hydrophobic groove of TamB, strongly suggesting its role in lipid transport as shown in parallel in a recent study.[42] Additionally, our simulations suggest that TamB alone may be able to facilitate lipid transfer if it can be trafficked to the OM and inserted such that the hydrophobic groove is continuous with the lower leaflet of the OM, although we cannot exclude the possibility that other proteins, including TamA, could play roles in offloading lipids at the OM. Finally, AF3 predictions of all 6 members of the AsmA-like family place palmitic acid molecules in the hydrophobic groove.

The TAM structures predicted using AF2 in this study and by others always place the complexation interface such that the OM segment of TamB completes the TamA β barrel. This is consistent with previous size-exclusion chromatography experiments and a recently published cryo-EM structure of the hybrid TamAB barrel.[14,20,42] This suggests TamA and TamB likely bind to each other at the OM mediated by interactions between β strands belonging to both proteins, and the TAM structure simulated here likely represents one plausible configuration of the two proteins in bacterial cells. Our use of a morphed AF2 model was necessary to enable correct placement of the TAM complex, consistent with the proposed orientation of TamA in the OM and TamB in the IM. Additional simulations using an unmorphed AF2 model show that lipid entry into the hydrophobic groove occurs irrespective of the model used, suggesting this is not an artifact of the model.

Interestingly, while lipid influx from the IM and traversal through the hydrophobic groove of TAM was rapid, lipids did not dissociate at the OM within the timescale of our simulations. In contrast, structural predictions of TamB alone suggest that it may be able to independently span the periplasm, and lipids readily fill the hydrophobic groove when it is embedded in the IM. Simulations of TamB in an alternate conformation, with its hydrophobic groove embedded in the OM, are stable and enable spontaneous phospholipid movement from the OM into the groove. Despite occurring in the reverse of the proposed transport direction, this suggests that lipid dissociation through this region may be possible. Together, these results suggest that TamB may facilitate phospholipid movement from the IM to the OM without TamA bound during this process. However, previous genetic knockout experiments have shown that TamA is required for TamB's roles in OM lipid homeostasis, with the same phenotypes being observed when tamA or tamB is knocked out in bacteria lacking other lipid transporters.[4,44]

There are several explanations that can account for these experimental and simulation data. Firstly, it's possible that lipid dissociation from TamB at the OM can occur while TamB is in complex with TamA in an alternative conformation not captured by our AlphaFold modeling or at timescales beyond what we can currently simulate. Secondly, it is also plausible that one or more additional accessory proteins aid in lipid offloading at the OM. Alternatively, our observations may be explained by a model in which TamA aids correct localization and folding of TamB at the OM, but where the two proteins are not associated during the lipid transfer process. This is consistent with recent work indicating that that disruption of β signal recognition or β1 interactions impairs folding by TAM, supporting a model in which the TamA-TamB interface may be disrupted during outer membrane protein assembly, although this has not been directly shown.[18]

Comparison of our simulations of TAM, YhdP, and YdbH reveal differences in the number of lipids that can be accommodated in the hydrophobic grooves of these proteins, with the wider groove of TamB permitting more than twice as many lipids than YhdP and YdbH to enter. These findings are consistent with the proposal that TamB may facilitate the trafficking of nascent OMPs to the OM via this hydrophobic groove, perhaps simultaneously allowing lipid transport.[18,45] YhdP is capable of spanning the periplasm alone and is longer than YdbH, which requires the aid of additional proteins to completely bridge the space between the IM and OM.[13,46] Despite this, fewer lipids were present in the groove of YhdP at the end of our simulations, owing to the narrow nature of the groove and the presence of a constriction in the groove in the middle of the protein in our predicted structure.

Our simulations of YhdP are consistent with previous findings that phosphate-containing molecules (ostensibly phospholipids) span the length of the YhdP groove, supporting its proposed role in lipid transport.[3–5,13] CG simulations spanning 5 μs by Cooper et al. captured on average one lipid entering the hydrophobic groove from the IM,[13] fewer than the 3–10 lipids we observed in the same timespan owing to our use of a different starting structure with the N-terminal loop displaced. Interestingly, spontaneous bilayer formation simulations conducted with the C-terminal segment of YhdP showed that the protein interacted only with the inner leaflet of the OM, and no lipid entry events were observed from the OM.[13] This finding might support the idea that an additional accessory protein could be required to offload lipids at the OM across the different transporters. Alternatively, the

YhdP conformation used or the timescale of the simulations could also account for this result.

Previous genetic studies showed that only one of TamB, YhdP, or YdbH is required for bacterial survival,[4,44] and the purpose of multiple redundant lipid transporters remains an open question. The possibility of differential phospholipid-substrate transport preferences between YhdP, TamB, and YdbH has been proposed to explain the existence of three redundant lipid transporters and differences in phenotypes when these proteins were knocked out.[47] These differences were based more strongly on the saturation of the fatty acyl chains rather than lipid headgroups, with YhdP proposed to transport cardiolipin and saturated lipids, while TamB was proposed to transport more unsaturated lipids. While the data from our simulations are insufficient to draw strong conclusions on this point, there does not appear to be any obvious selectivity for or against a specific lipid headgroup type by any of these proteins, nor a large difference between the types of lipids found in the hydrophobic groove when comparing between proteins, although we note that cardiolipin was not found in the hydrophobic groove of YhdP in any of our simulations. Further investigation of TamB, YhdP, and YdbH is necessary to determine whether these proteins exhibit differences in substrate specificity or could play distinct roles physiologically. In addition, much remains to be understood about how AsmA-like proteins work alongside the Pqi, Let, and Mla systems which are also implicated in bacterial phospholipid transport (reviewed in Giacometti et al.[48]). Further study is required to better understand the directionality of lipid transport, whether energy input is required, substrate specificity, and the broader physiological roles of these pathways.

Our findings offer new insights into lipid transport mechanisms in bacteria and the role of TamB in OM biogenesis. Given the essential nature of the AsmA-like proteins in bacterial survival, increasing our understanding of how they function is foundational to the development of antibiotics targeting these proteins.

## DATA AVAILABILITY

Simulation trajectories and AF2 models generated as part of this study are available on Zenodo (https://doi.org/10.5281/zenodo.19479271).

## ACKNOWLEDGMENTS

The authors thank Dr. Denisse Leyton and Dr. Matthew Doyle for helpful discussions. This research was undertaken with the assistance of resources and services from the National Computational Infrastructure (NCI), which is supported by the Australian Government.

## AUTHOR CONTRIBUTIONS

Y.L.: conceptualization, methodology, formal analysis, investigation, writing – original draft, writing – review and editing, visualization. B.C.: writing – review and editing, resources.

## DECLARATION OF INTERESTS

The authors declare no competing interests.

## SUPPLEMENTAL INFORMATION

Supplemental information can be found online at https://doi.org/10.1016/j.bpj.2026.05.019.

## REFERENCES

1. Silhavy, T. J., D. Kahne, and S. Walker. 2010. The Bacterial Cell Envelope. *Cold Spring Harbor Perspect. Biol.* 2:a000414.
2. Kumar, S., and N. Ruiz. 2023. Bacterial AsmA-Like Proteins: Bridging the Gap in Intermembrane Phospholipid Transport. *Contact.* 6:25152564231185931.
3. Grimm, J., H. Shi, …, T. J. Silhavy. 2020. The inner membrane protein YhdP modulates the rate of anterograde phospholipid flow in Escherichia coli. *Proc. Natl. Acad. Sci. USA.* 117:26907–26914.
4. Ruiz, N., R. M. Davis, …, T. B. YhdP. 2021. YdbH Are Redundant but Essential for Growth and Lipid Homeostasis of the Gram-Negative Outer Membrane. *mBio.* 12:e02714-21.
5. Douglass, M. V., A. B. McLean, and M. S. Trent. 2022. Absence of YhdP, TamB, and YdbH leads to defects in glycerophospholipid transport and cell morphology in Gram-negative bacteria. *PLoS Genet.* 18:e1010096.
6. Santarossa, C. C., Y. Li, …, G. Bhabha. 2026. LetA defines a structurally distinct transporter family. *Nature.* 651:1097–1106.
7. Ekiert, D. C., G. Bhabha, …, R. D. Vale. 2017. Architectures of Lipid Transport Systems for the Bacterial Outer Membrane. *Cell.* 169:273–285.e17.
8. Isom, G. L., N. Coudray, …, G. Bhabha. 2020. LetB Structure Reveals a Tunnel for Lipid Transport across the Bacterial Envelope. *Cell.* 181:653–664.e19.
9. Liu, C., J. Ma, …, L. Zhang. 2020. Cryo-EM Structure of a Bacterial Lipid Transporter YebT. *J. Mol. Biol.* 432:1008–1019.
10. Tang, X., S. Chang, …, H. Dong. 2021. Structural insights into outer membrane asymmetry maintenance in Gram-negative bacteria by MlaFEDB. *Nat. Struct. Mol. Biol.* 28:81–91.
11. Kolich, L. R., Y.-T. Chang, …, D. C. Ekiert. 2020. Structure of MlaFB uncovers novel mechanisms of ABC transporter regulation. *eLife.* 9:e60030.
12. Neuman, S. D., T. P. Levine, and A. Bashirullah. 2022. A novel superfamily of bridge-like lipid transfer proteins. *Trends Cell Biol.* 32:962–974.
13. Cooper, B. F., R. Clark, …, G. L. Isom. 2025. Phospholipid Transport Across the Bacterial Periplasm Through the Envelope-spanning Bridge YhdP. *J. Mol. Biol.* 437:168891.
14. Selkrig, J., K. Mosbahi, …, T. Lithgow. 2012. Discovery of an archetypal protein transport system in bacterial outer membranes. *Nat. Struct. Mol. Biol.* 19:506.
15. Heinz, E., C. J. Stubenrauch, …, T. Lithgow. 2016. Conserved Features in the Structure, Mechanism, and Biogenesis of the Inverse Autotransporter Protein Family. *Genome Biol. Evol.* 8:1690–1705.
16. Stubenrauch, C. J., R. S. Bamert, …, T. Lithgow. 2022. A noncanonical chaperone interacts with drug efflux pumps during their assembly into bacterial outer membranes. *PLoS Biol.* 20:e3001523.
17. Goh, K. J., C. J. Stubenrauch, and T. Lithgow. 2024. The TAM, a Translocation and Assembly Module for protein assembly and potential conduit for phospholipid transfer. *EMBO Rep.* 25:1711–1720.
18. Wang, X., S. B. Nyenhuis, and H. D. Bernstein. 2024. The translocation assembly module (TAM) catalyzes the assembly of bacterial outer membrane proteins in vitro. *Nat. Commun.* 15:7246.

19. Pronk, S., S. Páll, …, E. Lindahl. 2013. GROMACS 4.5: a high-throughput and highly parallel open source molecular simulation toolkit. *Bioinformatics.* 29:845–854.
20. McDonnell, R. T., N. Patel, …, A. H. Elcock. 2023. Atomic Models of All Major Trans-Envelope Complexes Involved in Lipid Trafficking in <em>Escherichia Coli</em> Constructed Using a Combination of AlphaFold2, AF2Complex, and Membrane Morphing Simulations. Preprint at bioRxiv. https://doi.org/10.1101/2023.04.28.538765.
21. Teufel, F., J. J. Almagro Armenteros, …, H. Nielsen. 2022. SignalP 6.0 predicts all five types of signal peptides using protein language models. *Nat. Biotechnol.* 40:1023–1025.
22. Jumper, J., R. Evans, …, D. Hassabis. 2021. Highly accurate protein structure prediction with AlphaFold. *Nature.* 596:583–589.
23. Varadi, M., D. Bertoni, …, S. Velankar. 2024. AlphaFold Protein Structure Database in 2024: providing structure coverage for over 214 million protein sequences. *Nucleic Acids Res.* 52:D368–D375.
24. Parrinello, M., and A. Rahman. 1981. Polymorphic transitions in single crystals: A new molecular dynamics method. *J. Appl. Phys.* 52:7182–7190.
25. Bussi, G., D. Donadio, and M. Parrinello. 2007. Canonical sampling through velocity rescaling. *J. Chem. Phys.* 126:014101.
26. Mirdita, M., K. Schütze, …, M. Steinegger. 2022. ColabFold: making protein folding accessible to all. *Nat. Methods.* 19:679–682.
27. Abramson, J., J. Adler, …, J. M. Jumper. 2024. Accurate structure prediction of biomolecular interactions with AlphaFold 3. *Nature.* 630:493–500. https://doi.org/10.1038/s41586-024-07487-w.
28. Huang, J., S. Rauscher, …, A. D. MacKerell. 2017. CHARMM36m: an improved force field for folded and intrinsically disordered proteins. *Nat. Methods.* 14:71–73.
29. Li, Y., J. Liu, and J. C. Gumbart. 2021. Preparing Membrane Proteins for Simulation Using CHARMM-GUI. *In* Structure and Function of Membrane Proteins. I. Schmidt-Krey and J. C. Gumbart, eds Springer US, pp. 237–251. https://doi.org/10.1007/978-1-0716-1394-8_13.
30. Evans, D. J., and B. L. Holian. 1985. The Nose–Hoover thermostat. *J. Chem. Phys.* 83:4069–4074.
31. Hess, B., H. Bekker, …, J. G. E. M. Fraaije. 1997. LINCS: A linear constraint solver for molecular simulations. *J. Comput. Chem.* 18:1463–1472.
32. Darden, T., D. York, and L. Pedersen. 1993. Particle mesh Ewald: An $N \cdot \log(N)$ method for Ewald sums in large systems. *J. Chem. Phys.* 98:10089–10092.
33. Gowers, R., M. Linke, …, O. Beckstein. 2016. MDAnalysis: A Python Package for the Rapid Analysis of Molecular Dynamics Simulations. Austin, pp. 98–105. https://conference.scipy.org/proceedings/scipy2016/oliver_beckstein.html.
34. McKinney, W. 2010. Data Structures for Statistical Computing in Python. Austin, pp. 56–61. https://doi.org/10.25080/majora-92bf1922-00a.
35. Virtanen, P., R. Gommers, …, SciPy 1.0 Contributors. 2020. SciPy 1.0: fundamental algorithms for scientific computing in Python. *Nat. Methods.* 17:261–272.
36. Humphrey, W., A. Dalke, and K. Schulten. 1996. VMD: Visual molecular dynamics. *J. Mol. Graph.* 14:28–33.
37. Pravda, L., D. Sehnal, …, M. Otyepka. 2018. MOLEonline: a web-based tool for analyzing channels, tunnels and pores (2018 update). *Nucleic Acids Res.* 46:W368–W373.
38. Schmidt, V., M. Sidore, …, J. N. Sturgis. 2019. The lipid environment of Escherichia coli Aquaporin Z. *Biochim. Biophys. Acta Biomembr.* 1861:431–440.
39. Gupta, K., J. A. C. Donlan, …, C. V. Robinson. 2017. The role of interfacial lipids in stabilizing membrane protein oligomers. *Nature.* 541:421–424.
40. Jayasekera, H. S., F. A. Mohona, …, M. T. Marty. 2025. Alanine Scanning to Define Membrane Protein–Lipid Interaction Sites Using Native Mass Spectrometry. *Biochemistry.* 64:1308–1316. https://doi.org/10.1021/acs.biochem.4c00717.
41. Shearer, J., D. Jefferies, and S. Khalid. 2019. Outer Membrane Proteins OmpA, FhuA, OmpF, EstA, BtuB, and OmpX Have Unique Lipopolysaccharide Fingerprints. *J. Chem. Theor. Comput.* 15:2608–2619.
42. Yang, B., R. Fan, …, C. Dong. 2026. Structural basis of outer membrane biogenesis by the TamAB translocase. *Nat. Commun.* 17:437.
43. Gruss, F., F. Zähringer, …, T. Maier. 2013. The structural basis of autotransporter translocation by TamA. *Nat. Struct. Mol. Biol.* 20:1318–1320.
44. Sposato, D., J. Mercolino, …, F. Imperi. 2024. Redundant essentiality of AsmA-like proteins in Pseudomonas aeruginosa. *mSphere.* 9:e0067723.
45. Josts, I., C. J. Stubenrauch, …, R. Grinter. 2017. The Structure of a Conserved Domain of TamB Reveals a Hydrophobic β Taco Fold. *Structure.* 25:1898–1906.e5.
46. Kumar, S., R. M. Davis, and N. Ruiz. 2024. YdbH and YnbE form an intermembrane bridge to maintain lipid homeostasis in the outer membrane of Escherichia coli. *Proc. Natl. Acad. Sci. USA.* 121:e2321512121.
47. Rai, A. K., K. Sawasato, …, A. M. Mitchell. 2024. Genetic evidence for functional diversification of gram-negative intermembrane phospholipid transporters. *PLoS Genet.* 20:e1011335.
48. Giacometti, S. I., M. R. MacRae, …, D. C. Ekiert. 2022. Lipid Transport Across Bacterial Membranes. *Annu. Rev. Cell Dev. Biol.* 38:125–153.

Biophysical Journal, Volume *125*

## Supplemental information

# Bacterial lipids traverse the hydrophobic groove of TamB

**Yiechang Lin and Ben Corry**

# Supplemental Information for

Bacterial lipids traverse the hydrophobic groove of TamB

Yiechang Lin,[1,2]* Ben Corry[1]

[1] Research School of Biology, Australian National University, Canberra, Australia 2601

[2] Present address: Center for Quantitative Biology, Peking University, China 100871

Yiechang Lin

Email: Yiechang.Lin@pku.edu.cn

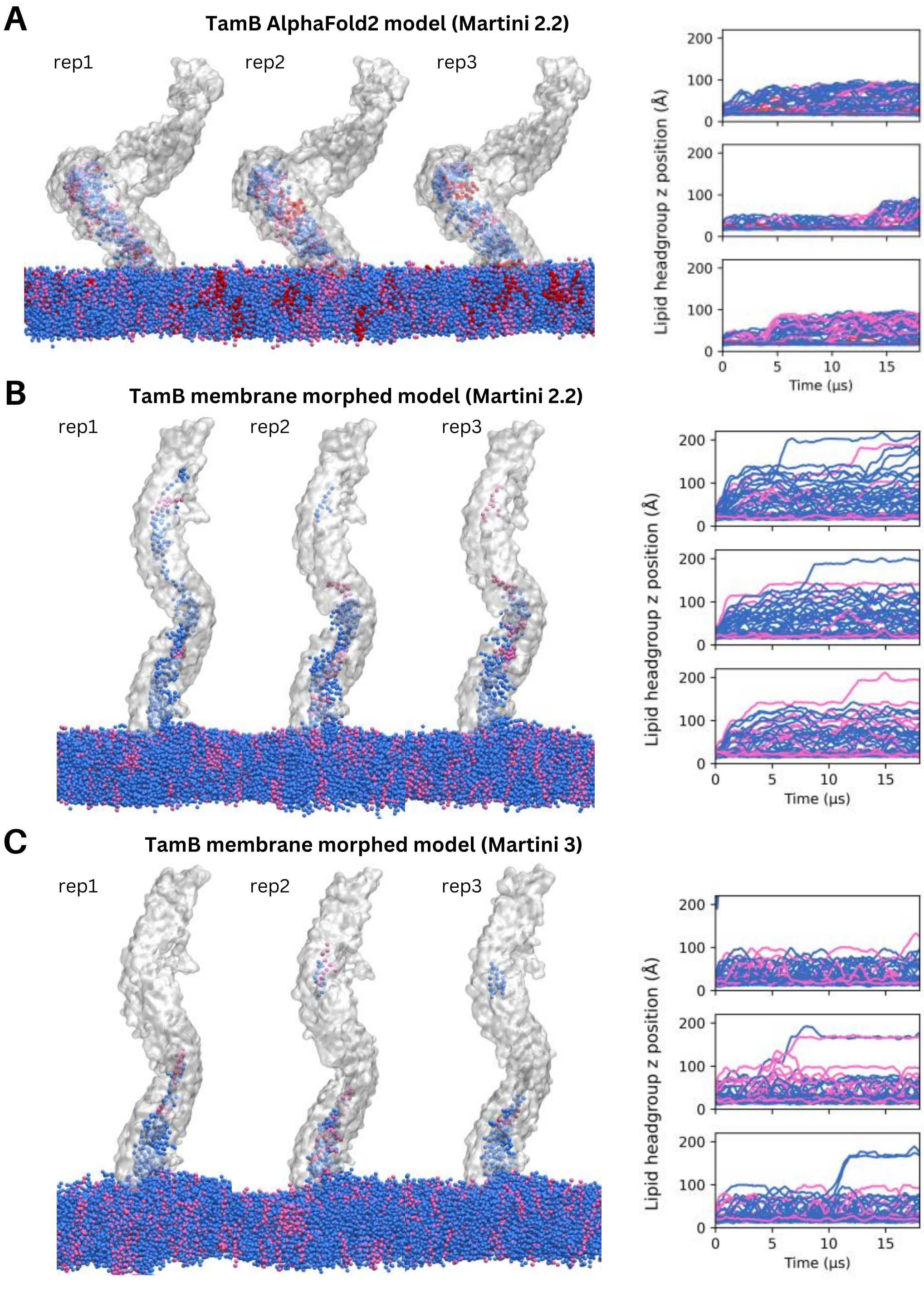


**Fig. S1. Lipid entry into TamB in single bilayer systems**

**(A)** Final frame snapshots (left) and lipid headgroup z position plots (right) for triplicate 18 µs simulations of the AlphaFold2 model of TamB, carried out using the Martini 2.2 forcefield.

**(B)** Final frame snapshots (left) and lipid headgroup z position plots (right) for triplicate 18 µs simulations of the membrane morphed model of TamB, carried out using the Martini 2.2 forcefield.

**(C)** Final frame snapshots (left) and lipid headgroup z position plots (right) for triplicate 18 µs simulations of the membrane morphed model of TamB, carried out using the Martini 3 forcefield.

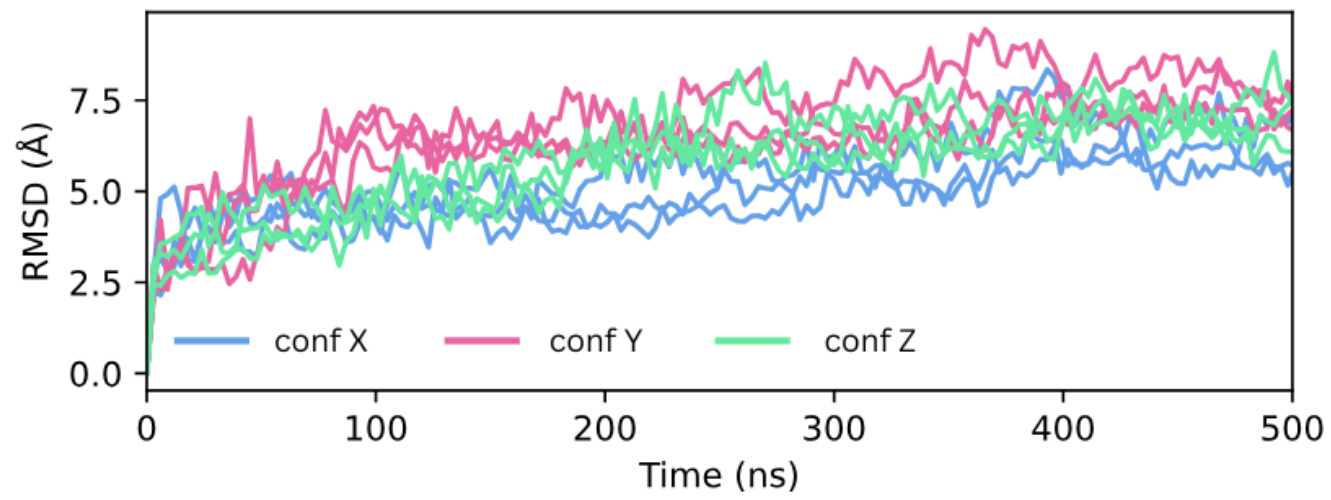


**Fig. S2.** Root mean square deviation (RMSD) plots for three conformations of the TamB OM segment in triplicate 500 ns all-atom simulation.

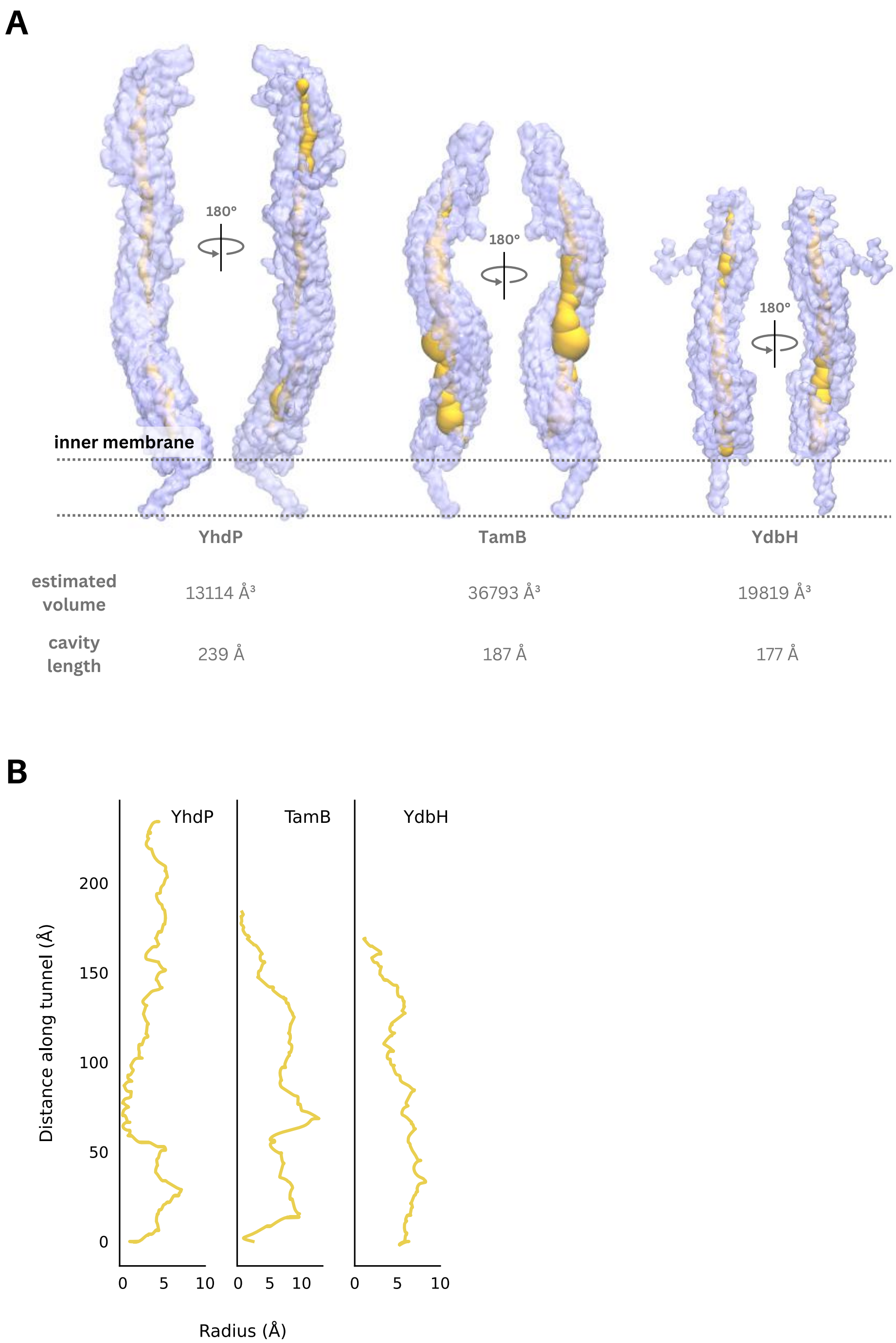


**Fig. S3. Cavity analysis of YhdP, TamB and YdbH**

**(A)** Visualisation of the internal cavities of YhdP, TamB and YdbH. Protein surface shown in transparent purple and tunnel through the cavity (yellow) as calculated by the MOLEonline server. The estimated volume and length of each tunnel is provided.

**(B)** 2D tunnel radius profile for each of YhdP, TamB and YdbH

**Movie S1 (separate file).** A 45 μs coarse grained simulation of TAM embedded in the E. coli inner membrane (IM) and outer membrane (OM) showing lipid movement from the IM into the hydrophobic groove of TamB. Lipids are colored by type (ReLPS – grey, POPE- blue, POPG – pink, CDL2 – red).
